# An Improved Diffusion Model for Generating Images of a Single Category of Food on a Small Dataset

**DOI:** 10.3390/foods15030443

**Published:** 2026-01-26

**Authors:** Zitian Chen, Zhiyong Xiao, Dinghui Wu, Qingbing Sang

**Affiliations:** 1 School of Artificial Intelligence and Computer Science, Jiangnan University, Wuxi 214122, China; 6233112010@stu.jiangnan.edu.cn (Z.C.);; 2 Engineering Research Center of Intelligent Technology for Healthcare, Ministry of Education, Wuxi 214122, China; 3 School of Internet of Things, Engineering Jiangnan University, Wuxi 214122, China

**Keywords:** food image generation, diffusion models, masked training, linear interpolation

## Abstract

In the era of the digital food economy, high-fidelity food images are critical for applications ranging from visual e-commerce presentation to automated dietary assessment. However, developing robust computer vision systems for food analysis is often hindered by data scarcity for long-tail or regional dishes. To address this challenge, we propose a novel high-fidelity food image synthesis framework as an effective data augmentation tool. Unlike generic generative models, our method introduces an Ingredient-Aware Diffusion Model based on the Masked Diffusion Transformer (MaskDiT) architecture. Specifically, we design a Label and Ingredients Encoding (LIE) module and a Cross-Attention (CA) mechanism to explicitly model the relationship between food composition and visual appearance, simulating the “cooking” process digitally. Furthermore, to stabilize training on limited data samples, we incorporate a linear interpolation strategy into the diffusion process. Extensive experiments on the Food-101 and VireoFood-172 datasets demonstrate that our method achieves state-of-the-art generation quality even in data-scarce scenarios. Crucially, we validate the practical utility of our synthetic images: utilizing them for data augmentation improved the accuracy of downstream food classification tasks from 95.65% to 96.20%. This study provides a cost-effective solution for generating diverse, controllable, and realistic food data to advance smart food systems.

## 1. Introduction

In the fields of food science and public health, image-based dietary assessment has emerged as a critical tool for precise nutritional monitoring [1,2]. Beyond health applications, high-fidelity food imagery plays a pivotal role in digital marketing, sensory evaluation, and automated food quality inspection, where visual appeal directly influences consumer perception and acceptability [3,4]. Unlike traditional self-reporting, automated recognition systems utilizing computer vision offer objective analysis. However, the robustness of these systems depends on large-scale datasets. A major bottleneck in food computing is the scarcity of high-quality, annotated data, particularly for long-tail or regional dishes [5,6]. Manual collection is labor-intensive, limiting the scalability of intelligent food systems.

Generative AI has emerged as a promising avenue for data augmentation. Early approaches largely relied on Generative Adversarial Networks (GANs). In the food domain, Han et al. introduced the Multi-Component Pizza Generator (MPG) [7] based on StyleGAN2 [8] to generate pizza images with specified ingredients. Similarly, CookGAN [9] pioneered a recipe-to-food approach, synthesizing images from cooking steps. Fu et al. [10] further tackled inter-class entanglement using StyleGAN3 [11]. However, despite their high-resolution, GAN-based methods often suffer from mode collapse and lack fine-grained semantic controllability, making it difficult to precisely alter specific ingredients without distorting the global structure [12].

More recently, diffusion models have surpassed GANs in generation quality and diversity. Originating from the Denoising Diffusion Probabilistic Models (DDPMs) framework [13], these models generate images by progressively refining noise. As highlighted by Karras et al. [14], the modular nature of diffusion allows for flexible improvements. While traditional models [15,16,17] relied on U-Net architectures, the state of the art has shifted towards Transformer-based backbones [18]. Xie et al. proposed the Diffusion Transformer (DiT) [19], which achieved benchmark-breaking performance on ImageNet. Building on this, Zheng [20] and Gao [21] integrated masked training [22] into DiT, predicting unmasked patches to reduce training costs while improving FID scores [23].

Despite these architectural advancements, applying generic diffusion models to the food domain remains challenging. Generic models are typically “data-hungry” and fail to learn robust distributions from small-scale food datasets. Recent attempts to bridge this gap, such as Markham’s FoodFusion [24] and Yue Han’s conditional diffusion models [25], have made progress but are often limited by their reliance on single data modalities (e.g., image-only or text-only conditioning), restricting their ability to fully utilize heterogeneous ingredient data. Currently, there is a lack of frameworks specifically designed for high-fidelity, single-category food synthesis that can operate effectively under data scarcity while maintaining semantic consistency.

To address this gap, we propose a novel Ingredient-Aware Diffusion Model. Our primary objective is to develop a data-efficient framework capable of synthesizing photorealistic images for specific food categories using limited training samples. We adopt the MaskDiT architecture for its efficiency in capturing local textural details. A key innovation is the adaptive flexibility to handle heterogeneous food data. We design two plug-and-play modules: the label and Ingredients Encoding (LIE) module and the Cross-Attention (CA) mechanism. These modules function dynamically: in “Ingredient-Aware Mode,” they explicitly align visual features with ingredient semantics, facilitating fine-grained control over dish composition and appearance; in “Label-Conditional Mode,” the framework adapts to label-only data. This dual-mode capability maximizes utility across disparate data sources.

Furthermore, to eliminate inter-class feature entanglement, a common issue where visual traits of different dishes bleed into one another, we adopt a targeted Single-Category Training strategy. By focusing on a single category at a time, our framework captures unique structural and textural distributions, ensuring superior realism. To stabilize training on small-scale datasets, we incorporate a linear interpolation strategy [26], enabling robust convergence with as few as 1000 samples. We validate our method via FID on Food-101 [27] and VireoFood-172 [28] and a downstream food classification experiment. Results demonstrate that our synthetic data significantly boosts the recognition accuracy of classifiers trained on limited real data, offering a cost-effective solution for constructing high-quality food databases.

The main contributions of this work are summarized as follows:

1. Data-Efficient Framework: We propose a novel Ingredient-Aware Diffusion Model integrating linear interpolation into MaskDiT. This approach stabilizes training dynamics on small-scale datasets, enabling high-fidelity generation with limited samples.
2. Adaptive Semantic Modeling: We design plug-and-play LIE and CA modules to explicitly align visual features with ingredient semantics. These provide flexibility to handle heterogeneous data, switching adaptively between recipe-driven and label-driven modes.
3. Practical Utility Validation: We demonstrate the value in food informatics by verifying effectiveness in data augmentation. Utilizing our synthetic images improved downstream food classification accuracy, offering a viable solution to data scarcity in smart food systems.

## 2. Method

**Figure 1:**
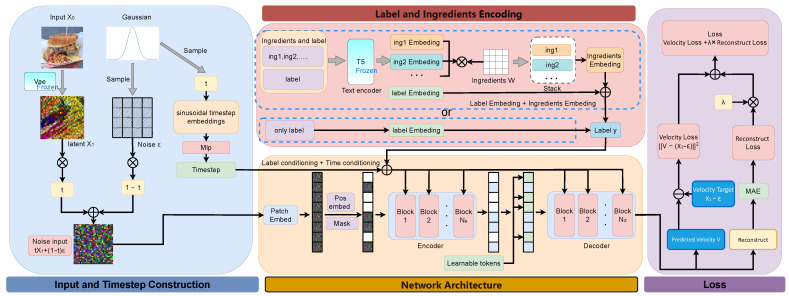
The architecture of the proposed network is illustrated. In the figure, × represents multiplication and + represents addition.

In this section, we introduce the improved diffusion model. To address class entanglement among food items, we adopt a single-class generation approach, enhancing performance on single-class tasks through linear interpolation. This method, combined with adapted speed prediction [29], enables the model to excel on small single-class datasets, making it suitable for real-world food image generation tasks. Our overall framework, illustrated in Figure 1, consists of four steps: First, we construct input and time steps by sampling from a Gaussian distribution. Second, we use ingredient information for more effective conditional control. The third step involves predictions using the MaskDiT module and cross-layer attention. Finally, we determine the prediction target through the loss function.

### 2.1. Input and Timestep Construction

In diffusion models, the noise is time-dependent and follows a Gaussian distribution. The diffusion model gradually adds Gaussian noise to the real data *X*, causing the images to ultimately converge to a normal distribution. The specific formula is as follows:

(1) $$X\left(t\right)=\alpha_{t}X+\sigma_{t}\varepsilon$$

In the equation, $\alpha_{t}$ and $\sigma_{t}$ are hyperparameters.

To effectively apply the diffusion model to food image generation, we introduce a new interpolation method and pre-training tasks to replace the EDM module in the original MaskDiT. We add noise to the VAE-encoded latent space images through linear interpolation [26] and train using velocity prediction. As shown in the blue section of Figure 1, we first map the original image $X_{0}$ to the latent space with a frozen-weight VAE encoder [30], producing the latent space image $X_{1}$. We then implement a uniform change, rewriting Equation (1) as follows:

(2) $$X\left(t\right)=tX_{1}+\left(1-t\right)\varepsilon \;$$

The value of t ranges from 0 to 1. In this process, the model first samples time and noise from a Gaussian distribution, then maps *t* to discrete time steps using sinusoidal time step encoding and MLP layers. These time steps act as temporal control conditions within the MaskDiT network architecture, facilitating conditional control during the denoising process. Meanwhile, $X_{1}$ obtained after adding noise as described in Equation (2), is also input into the MaskDiT network.

### 2.2. Label and Ingredients Encoding

We designed the LIE module to improve the semantic representation of class labels. As illustrated in the blue dashed section above the red area in Figure 1, the LIE module comprises a frozen-weight T5 text encoder [31] and an adaptive weight matrix. The T5 encoder encodes text information for further computations, with its parameters remaining fixed during updates. The adaptive weight matrix regulates the proportions of different ingredients in the food. We denote the class label as $\varphi_{label}$ and the ingredient prompt as $\varphi_{ing_{m}}$. The specific process can be represented as follows:

(3) $$\varphi_{label}^{\prime }=\varphi_{label}+\sum_{m=1}^{M}W_{m}*\varphi_{ing_{m}}$$

Here, *M* represents the total number of ingredients, and $W_{m}$ denotes the importance of ingredient *m* in the target food. We accumulate the results of multiplying each ingredient prompt by the weight matrix *W*, and then add this to the class label prompt to obtain the new class label $\varphi_{label}^{\prime }$. In this way, similar food labels exhibit subtle differences due to variations in ingredient composition, further providing a more refined control for food image generation.

As shown in the blue dashed section below the red area in Figure 1, when ingredient prompts are absent, we can rely solely on class label information for conditional control, ensuring the model remains flexible.

### 2.3. Network Architecture

The network architecture is illustrated in the yellow area of Figure 1. After inputting the noisy image $X_{t}$, the image is divided into *N* patches, to which position encoding is added. Several patches(n×patch) are then randomly removed based on a fixed masking ratio. The remaining uncovered patches((N−n)×patch) are denoised using an encoder with the same structure as the original DiT to predict their values. After processing the uncovered patches, a learnable parameter fills in the masked portions, resulting in a new image $X_{t}^{\prime }$. This $X_{t}^{\prime }$ is then input into a lightweight decoder to generate image reconstruction predictions for the masked patches and value predictions for the uncovered patches.

**Figure 2:**
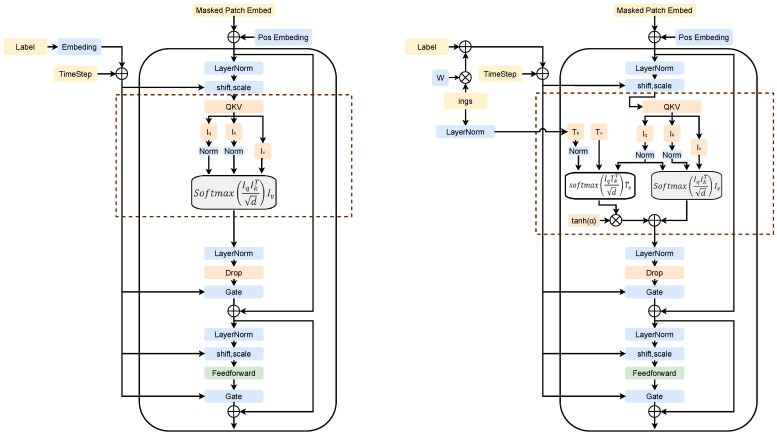
Detailed architecture of the transformer blocks. (**Left**): The standard MaskDiT block used for label-only generation. (**Right**): Our proposed Ingredient-Aware block integrating the Cross-Attention (CA) mechanism. Here, the ingredient embeddings (*W*) are injected into the visual features through the cross-attention layer [32], which is modulated by a zero-initialized gate [33,34] to adaptively control the fusion of recipe semantics.

As illustrated on the right side of Figure 2, we modified the encoder and decoder in MaskDiT to leverage diverse ingredient text information for generating food images. A zero-initialized gating mechanism adjusts the weight of the ingredient text information in the attention computation, represented by the following formula:

(4) $$\;\mathrm{attn}\;=\mathrm{softmax}\left(\frac{I_{q}I_{k}^{T}}{\sqrt{d}}\right)I_{v}+\tanh\left(\alpha \right)*\mathrm{softmax}\left(\frac{I_{q}T_{k}^{T}}{\sqrt{d}}\right)T_{v}$$

In this context, $T_{k}$ and $T_{v}$ represent the keys and values of the text, respectively, while α is a learnable parameter initialized to zero at the start of training. To ensure consistency when handling ingredient information, we apply Equation (4) for attention computation in both the encoder and decoder.

When only class label information and the time step are used as control conditions, the MaskDiT block retains the original model’s attention computation settings, as shown on the left side of Figure 2.

### 2.4. Loss Function

To better utilize linear interpolation, we replaced the original score prediction S(X(t),t) in MaskDiT with a prediction of the velocity field V(X(t),t). As illustrated in the left region of the purple section in Figure 1, we perform velocity prediction for the uncovered parts. During the training process, we predict the velocity using a neural network to get as close as possible to this objective. The velocity loss for the uncovered parts is calculated as follows:

(5) $$L_{v}=\int_{0}^{1}E_{X_{0}∼\;\mathrm{data}\;,\varepsilon ∼N\left(0,1\right)}\left[{∥\left(X_{0}-\varepsilon \right)-V\left(x\left(t\right),t\right)∥}^{2}\right]dt$$

While predicting the velocity of the uncovered patches, we also perform image reconstruction for the masked parts to recover global information lost due to the occlusion in the original image. As shown in the right region of the purple section in Figure 1, we use the MAE reconstruction loss [22] as the loss function. The reconstruction loss for the masked parts is computed as follows:

(6) $$L_{MAE}=\frac{1}{n}\sum_{i=1}^{n}{∥{\hat{y}}_{i}-y_{i}∥}^{2}$$

In MaskDiT, a weight coefficient $L_{MAE}$ is multiplied with λ(0<λ<1) when calculating the loss, in order to reduce the impact of $L_{MAE}$ during the training process and ensure that $L_{v}$ dominates most of the time. The final optimization objective can be expressed as:

(7) $$L=L_{v}+\lambda *L_{MAE}$$

## 3. Experiments and Discussion

### 3.1. Experimental Setup

We evaluated our model on four distinct datasets: Food-101 (“Hamburger” category and “Chicken Wings” category) and Food-172 (“Braised Pork” category and “Sweet mung bean soup” category). Performance was quantified using the Fréchet Inception Distance (FID). FID calculates the distance between feature vectors of real and generated images; a lower FID score indicates that the generated images are more realistic and closer to the real data distribution. Training was conducted with a batch size of 16 following the MaskDiT protocol, using AdamW optimizer [35]. The process consisted of two stages: (1) Diffusion Training: 100 K steps with a 50% masking ratio and 0.1 label dropout; (2) Velocity Fine-tuning: 14 K steps with a 0% masking ratio to optimize the final generation quality.

### 3.2. Quantitative Evaluation

We compared our improved model against recent leading approaches, including DiT [19], stable- diffusion-v1-4 [36], and CookGALIP [37]. We utilized the Fréchet Inception Distance (FID) as the primary metric, where a lower score indicates better image quality and higher diversity close to the real data distribution.

Performance on Standard Benchmarks (Food101). As shown in Table 1a, we first evaluated the methods on “Hamburger” and “Chicken Wings” from the widely used Food101 dataset. Our approach consistently surpasses both the CNN-based LDM and the Transformer-based DiT. Notably, on the “Hamburger” category, our method achieves a remarkable FID of 10.93, compared to 23.82 for DiT. This confirms that our proposed modules provide superior generation fidelity on standard objects.

Performance on Regional Cuisines (Food172). To test the model’s robustness on more complex, regional dishes, we evaluated it on “Braised Pork” and “Sweet mung bean soup” from the Food172 dataset (Table 1b). Our model achieves an FID of 17.26 on “Braised Pork,” significantly outperforming CookGALIP (37.15) and Finetuned LDM (29.58). These results indicate that our “digital cooking” strategy effectively captures the fine-grained visual characteristics of specific regional foods that baselines often fail to reproduce.

**Figure 3:**
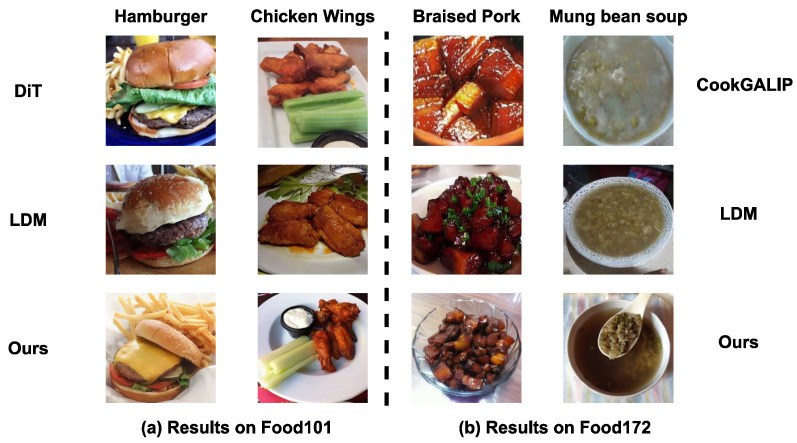
We visualize results for four representative categories: Hamburger and Chicken Wings from the Food101 dataset, and Braised Pork and Sweet Mung Bean Soup from the Food172 dataset.

Figure 3 compares the generation quality trained on small-scale dataset. Our model generates photorealistic images with rich details even when training data is scarce. Notably, for the regional Sweet Mung Bean Soup, our model preserves the distinct granular texture that baselines fail to reproduce, demonstrating exceptional generalization capabilities on small datasets.

### 3.3. Validation of Recipe-Consistent Synthesis

**Figure 4:**
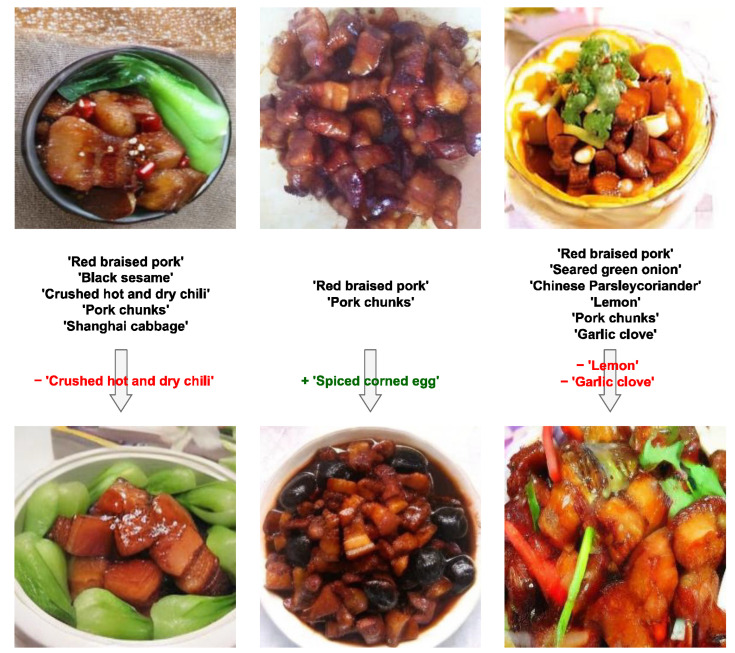
Examples of food images generated by the model using different recipes on the braised pork dataset from Food 172.

To evaluate the fidelity of our model in translating textual ingredient lists into corresponding visual structures, we conducted qualitative experiments on ingredient manipulation. As shown in Figure 4, the model demonstrates precise semantic control over food composition while maintaining photorealistic textures.

1. Ingredient Removal: In the first column, removing “Crushed hot and dry chili” from the text prompt results in a dish where the chili texture is absent, yet the glossy, gelatinous texture of the braised pork is preserved. This indicates that the model successfully disentangles specific ingredient features from the global dish appearance.
2. Ingredient Addition: In the second column, adding “Spiced corned egg” generates a geometrically consistent egg integrated naturally with the pork chunks.
3. Complex Modification: The third column shows simultaneous ingredient removal, proving the model’s robustness in handling complex recipe alterations.

These results validate that our LIE module effectively establishes a robust mapping between textual ingredient descriptors and visual feature representations. This ensures that the generated images are not only visually plausible but also compositionally accurate, meeting the strict standards required for applications such as standardized visual menu creation and dietary guideline illustration.

### 3.4. Impact of Using Generated Images on Food Classification

To validate the practical utility of our synthetic data in downstream tasks, we designed a few-shot binary classification experiment. The goal is to determine whether augmenting a limited real dataset with our synthetic images improves classification performance compared to using real data alone or data generated by other state-of-the-art models. Experimental Setup. We focused on two distinct categories: “Hamburger” and “Chicken Wings,” employing a pre-trained ResNet50 [38] as the classifier. To ensure the robustness of our results, all experiments were repeated with three different random seeds, and we report both the mean values and standard deviations. The dataset was split as follows:

1. Training Set (Few-shot Baseline): A limited set of 200 real images (100 per class).
2. Augmented Training Sets: The baseline set augmented with 400 synthetic images (200 per class) generated by DiT, Finetuned Latent Diffusion, and our proposed method, respectively.
3. Test Set: A large-scale held-out set of 1600 real images (800 per class) to ensure statistical reliability.

Quantitative Results. The classification performance (Accuracy and Specificity) is reported in Table 2. The “Real Only” baseline achieved a high accuracy of 95.65%, indicating the strong feature extraction capability of ResNet50. Interestingly, augmenting the dataset with images from DiT and Finetuned Latent Diffusion resulted in a performance drop (95.09% and 95.39%, respectively). This phenomenon is likely due to the data-hungry nature of these baselines. When trained on limited datasets, they struggle to learn generalized representations, often producing artifacts or distribution shifts that introduce noise to the classifier.In contrast, Our method was the only method to achieve a positive gain, improving the accuracy to 96.20% and Specificity to 96.28%. Furthermore, our method demonstrated the lowest standard deviation (±0.48), indicating superior training stability even under data-constrained conditions.

**Figure 5:**
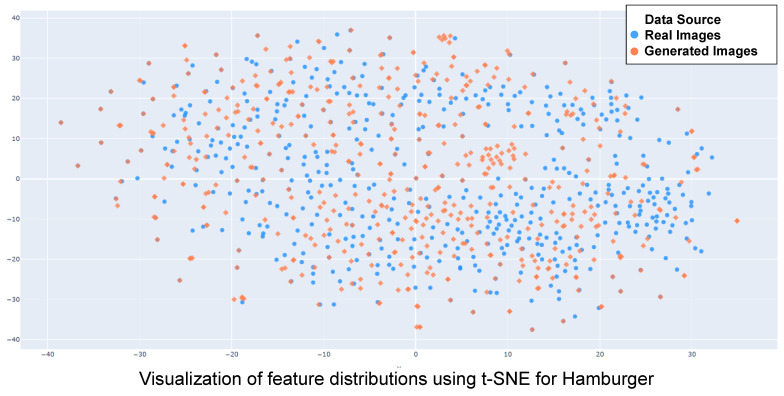

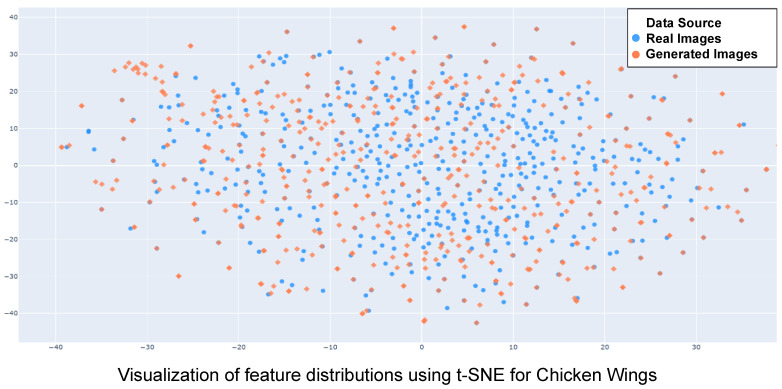
t-SNE visualization of feature distributions comparing real and generated images. (**Top**) Distribution for Hamburger; (**Bottom**) Distribution for Chicken Wings. For both categories, the generated samples are indistinguishably mixed with the real data manifold, demonstrating high diversity and effective coverage without mode collapse.

Distribution Analysis. We visualized the feature distributions of the “Hamburger” and “Chicken Wings” classes using t-SNE (as shown in Figure 5). The visualization reveals that our generated samples (orange points) are statistically well-aligned with the real samples (blue points) and effectively fill the sparse manifold gaps inherent to small-scale datasets. Unlike baseline methods that may generate out-of-distribution samples due to overfitting on limited data, our strategy ensures semantic consistency. This allows the classifier to establish a more robust decision boundary without overfitting to synthetic artifacts, thereby confirming that the performance gains stem from meaningful data diversity rather than memorization.

### 3.5. Ablation Studies

To thoroughly evaluate the contribution of each proposed component—Linear Interpolation, LIE module, and CA mechanism—we conducted comprehensive ablation studies.

Effectiveness of Linear Interpolation Strategy. We first investigate the impact of the Linear Interpolation strategy. As the backbone of our framework is adapted from MaskDiT, we compare our method directly against the original MaskDiT under identical training conditions (100 K steps). As shown in Table 3, the introduction of Linear Interpolation significantly stabilizes the training process on small-scale datasets, drastically reducing the FID score from 62.45 to 10.93. This demonstrates that the standard EDM training objective in the original MaskDiT is less effective for data-scarce food domains, whereas our velocity-prediction-based interpolation provides superior convergence.

Effectiveness of Semantic Modules (LIE & CA). Next, we evaluate the semantic control modules: Label and Ingredients Encoding (LIE) and Cross-Attention (CA). Using the model with Linear Interpolation as the new baseline, we sequentially incorporate LIE and CA to assess their added value. Table 4 presents the results on the Food-172 Braised Pork dataset. The addition of the LIE module improves FID from 22.16 to 17.63, proving its ability to encode recipe logic. Further integration of the CA mechanism optimizes the score to 17.26, confirming that explicit visual-semantic alignment enhances fine-grained texture generation.

## 4. Discussion

The intersection of generative AI and food informatics presents new opportunities for nutritional science and digital food marketing. Our study addresses two critical challenges in this domain: data scarcity and semantic consistency.

Ensuring Nutritional Accuracy: While data-hungry models like DiT [19] and Latent Diffusion [13] degrade in data-scarce scenarios, our Linear Interpolation strategy stabilizes training, aligning with flow matching insights [17,26,39] regarding simplified generative trajectories. Crucially, our Ingredient-Aware architecture prevents ingredient “hallucination,” ensuring the precise visual-textual alignment essential for downstream applications like automated calorie estimation.

Solving the Data Bottleneck: Traditional food databases [27,28] often lack diversity for local cuisines [4]. Our proposed framework, validated by the classification experiment (Section 3.4), demonstrates that synthetic data can effectively substitute for real data in training recognition systems [40,41]. This offers a cost-effective pathway to digitize and preserve visual data for traditional or rare food cultures.

Limitations and Future Work: While our Single-Category Training strategy ensures high fidelity, it limits the model to intra-category generalization, precluding zero-shot synthesis of unseen categories. A notable failure mode arises when prompts include out-of-distribution ingredients; in such cases, the LIE module may fail to retrieve corresponding visual features, resulting in ingredient omission or generic textures. Future work will aim to develop a unified multi-category model and integrate nutritional metadata to create fully annotated synthetic datasets.

Efficiency and Stability: Despite the addition of LIE and CA modules, computational overhead is minimized as the T5 encoder utilizes frozen weights, adding no trainable parameters. Furthermore, our Linear Interpolation strategy ensures training stability in low-data regimes (Table 3). By simplifying the generative trajectory, it effectively mitigates convergence issues typically associated with data-scarce training.

## 5. Conclusions

In this study, we addressed the critical bottleneck of data scarcity in food computing by proposing a data-efficient, high-fidelity food image synthesis framework. By integrating a Linear Interpolation strategy into the MaskDiT architecture, our method enables stable convergence and high-quality generation on small-scale datasets. We further introduced adaptive Label and Ingredients Encoding (LIE) and Cross-Attention (CA) modules, which facilitate flexibility between recipe-driven and label-driven modes while ensuring ingredient-visual consistency. Validated on Food-101 and VireoFood-172, our approach not only achieves state-of-the-art visual quality but also demonstrates significant practical utility: augmenting training sets with our synthetic images improved downstream food classification accuracy from 95.65% to 96.20%. Looking ahead, we plan to integrate nutritional metadata (e.g., calories, macronutrients) into the generation process, moving towards a comprehensive, semantically annotated synthetic food database for the next generation of smart food systems.

## Figures and Tables

**Table 1:** Quantitative comparison of FID scores. We evaluate our method against baselines on (**a**) the standard Food101 dataset and (**b**) the complex regional Food172 dataset. The symbol ↓ indicates that lower values denote better performance.

**(a) Results on Food101 Dataset**		
Method	**Hamburger**	**Chicken Wings**
	**FID**↓	**FID**↓
DiT [19]	23.82	25.80
Finetuned Latent Diffusion [36]	35.27	22.36
**Ours**	**10.93**	**19.81**
**(b) Results on Food172 Dataset**		
Method	**Braised Pork**	**Sweet Mung Bean Soup**
	**FID**↓	**FID**↓
CookGALIP [37]	37.15	44.98
Finetuned Latent Diffusion [36]	29.58	35.70
**Ours**	**17.26**	**24.14**

**Table 2:** Few-shot classification performance with ResNet50 using real 100-shot and synthetic images.

Method	Specificity (%)	Accuracy (%)
Real (Baseline)	95.76 (±1.11)	95.65 (±1.28)
DiT	95.31 (±0.90)	95.09 (±1.08)
Finetuned LDM	95.53 (±1.80)	95.39 (±1.04)
**Ours**	**96.28 (±0.45)**	**96.20 (±0.48)**

**Table 3:** We compared the FID scores of our model with those of the original MaskDiT.

Method (Train Steps)	FID ↓
MaskDiT (100 K steps)	62.45
Ours (100 K steps)	10.93

**Table 4:** The performance of the two modules on the braised pork dataset from food172.

Architecture	FID ↓
Baseline	22.16
Baseline/LIE	17.63
Baseline/LIE/CA	17.26
LIE (without W)	23.82

## Data Availability

The original contributions presented in this study are included in the article. Further inquiries can be directed to the corresponding author.
